# Return to work rates of workers compensation patients with lumbar radiculopathy following epidural steroid injection

**DOI:** 10.1016/j.inpm.2026.100736

**Published:** 2026-02-05

**Authors:** Sean Pickard, David Speach, Kurt Hauber, Wesley Edwards, Andrea Baran, Alyssa Fedorko

**Affiliations:** a University of Rochester Medical Center, 601 Elmwood Avenue, Rochester, NY, 14642, USA; b Corewell Health Greenville Hospital Multispecialty Center, 707 S Greenville West Drive, Greenville, MI, 48838, USA

## Abstract

**Background:**

Low back injury is one of the leading causes of work-related injuries, disability, and lost productivity. Patients with lumbar radiculopathy are a subgroup of patients within work related low back injury. To our knowledge, there are few studies that specifically assessed the relationship between treating radiculopathy with an epidural injection and return to duty for injured workers. Treatments such as lumbar epidural steroid injection (ESI) that potentially expedite safe return to work could have cost-saving benefits by reducing the need for spine surgery while ameliorating an injured worker's pain and suffering.

**Objective:**

The objective of the study is to estimate the return-to-work rate in worker's compensation patients treated at a single academic site who were diagnosed with work related low back injury with lumbar radicular pain and treated with lumbar ESI.

**Methods:**

Electronic medical record data was obtained from the University of Rochester Clinical & Translational Science Institute in a retrospective review. Patients evaluated in the departments of Physical Medicine and Rehabilitation or Orthopaedics at a tertiary care medical center from January 1, 2012 to October 31, 2023. with at least one visit for lumbar radiculopathy, lumbar spinal stenosis and lumbar disc herniation were included in the study. Return to work rates were estimated from all workers treated with a lumbar ESI. Patient age, gender and number of injections were evaluated for their effect on RTW rate.

**Results:**

23 of 222 patients treated with a lumbar ESI returned to work, this totaled 10.4 % of the subgroup (95 % CI: 6.7 %–15.1 %). There was insufficient evidence of an association between gender and return to work following treatment with a lumbar ESI (OR for male = 1.09, 95 % CI 0.44–2.69, p = 0.86). Similarly, there was no association between the number of injections and return to work. (OR for 3+ injections compared to 1–2 injections 0.73, 95 % CI 0.30–1.80, p = 0.49). However, we observed a decrease in likelihood of returning to work as age increases in patients treated with ESI (OR per 10 years of age = 0.51, 95 % CI 0.36–0.73, p = 0.0002).

**Conclusion:**

Return to work rates in worker's compensation patients were 10.4 % after treatment with lumbar ESIs. It appears that even with treatment of lumbar ESIs, worker's compensation patients with lumbar radiculopathy had a low chance of returning to work.

## 1 Introduction

**Diagram 1:**
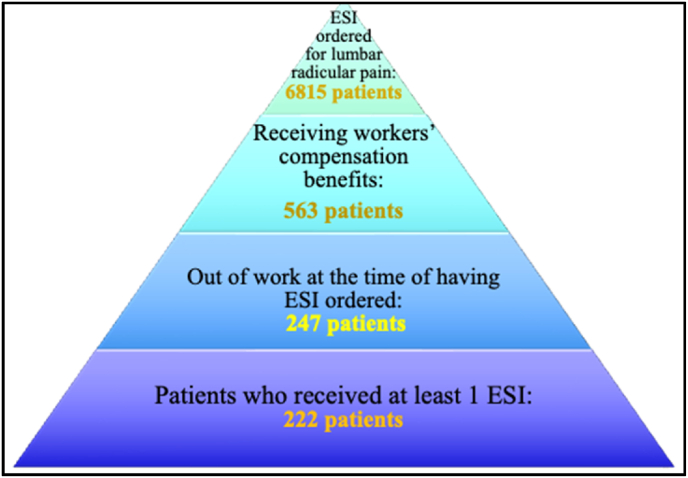

**Graph 1:**
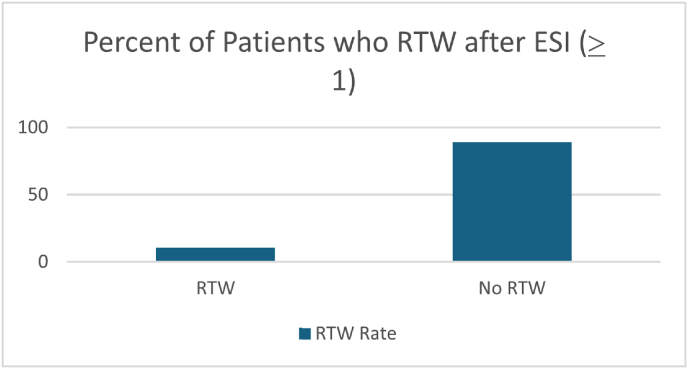
RTW Rates in patients who received at least 1 ESI.

Low back injury is one of the leading causes of work-related injuries, disability, and lost productivity. According to the U.S. Bureau of Labor Statistics, in 2016 musculoskeletal disorders of the back accounted for nearly 40 % of all work-related musculoskeletal injuries [1] with an average length of disability of 102 days [2]. Patients with longer lengths of time away from work contribute more substantially to overall claims cost with about 10 % of cases accounting for nearly 86 % of total costs [2]. The presence of low back pain (LBP) is a global issue and noted to cause a significant burden on healthcare and the economy. A recent study indicated that annual costs of LBP are up to $2.2 billion on a population level, with indirect costs of $1.7 billion due to work absenteeism [3] (see Table 1, Diagram 1, Graph 1).

**Table 1:** Patient characteristic Table (N = 247).

Continuous Variables	Median (IQR)
Age	50 (37–58)
Categorical Variables	
Injection	
No	25 (10.1 %) – Excluded from RTW analysis
Yes	222 (89.9 %)
RTW	
No	199 (89.6 %)
Yes	23 (10.4 %)
Gender	
Female	95 (38.5 %)
Male	152 (61.5 %)

Work related low back injury with lumbar radicular pain are a subgroup of patients that are commonly treated throughout the United States. Treatment of lumbar radiculopathy tends to be more effective than treatment of axial low back pain. There are multiple references, guidelines and systematic reviews available to assist with the diagnosis and management of lumbar radiculopathy [4]. Image guided lumbar epidural steroid injections (ESI) are one of several treatments available for lumbar radiculopathy. ESIs are utilized for lumbar radiculopathy when initial conservative treatments have failed and are often performed in the hope of avoiding spine surgery.

Prior literature and guidelines evaluating the effectiveness of lumbar ESI for the management of lumbar radiculopathy do not describe clear return to work outcomes for injured workers [5,6]. Lumbar ESIs used to treat lumbar radiculopathy may be ineffective, have rare catastrophic complications and may increase the cost of care [7]. There can be an onerous administrative burden to obtain prior authorization for a lumbar ESI under the workers compensation system. New York State provides detailed guidelines for the evaluation and treatment of lumbar radiculopathy [8]. A single study evaluating injured workers with acute low back pain demonstrated a positive influence on return-to-work (RTW) rates when clinicians adhered to the guidelines [9]. Treatments such as lumbar ESI that potentially expedite safe return to work could have cost-saving benefits by reducing the need for spine surgery while ameliorating an injured worker's pain and suffering. As our providers noticed that RTW rates appeared low, the goal of our study was to determine the RTW rate in our single academic center for patients in that specific subgroup (work related low back injury with lumbar radicular pain) who were treated with lumbar ESI in just under a 10 year span.

## 2 Methods

The study was approved by an institutional review board for a retrospective minimal risk analysis. Inclusion criteria for the study were patients 18 years and older injured at work with a diagnosis of lumbar radiculopathy or radiculopathy. Electronic medical record data was obtained from the University of Rochester Clinical & Translational Science Institute. Patients evaluated in the departments of Physical Medicine and Rehabilitation or Orthopaedics at a tertiary care medical center from January 1, 2012 to October 31, 2023 with at least one visit for lumbar radiculopathy, lumbar spinal stenosis and lumbar disc herniation were included in the study. Patients were then categorized into work injury related low back pain vs non-work injury related back pain. Additional data review identified the subgroup of patients treated with a lumbar ESI using the CPT codes: 64483/64484/62311. Patients who met inclusion for the study were categorized into a final subgroup of work-related injury using the payor information assigned to the patient's claim. Patient age, gender, number of epidural injections, and return to work were obtained by manual review of each patient's medical record. Data was de-identified during statistical analysis.

Injured workers with lumbar radiculopathy included in the study were evaluated and treated in accordance with the state workers compensation medical treatment guidelines [8]. The state medical treatment guidelines utilize published references and systematic reviews for managing lumbar radiculopathy, prioritizing non-surgical treatment of radiculopathy over spine surgery. Lumbar epidural steroid injections were offered to injured workers with radiculopathy when medications, physical therapy or chiropractic care failed to improve pain and function.

Patient characteristics were summarized using medians and interquartile ranges (IQR) for continuous variables and counts and proportions for categorical variables. Return to work rates estimated recorded in all workers treated with a lumbar ESI. Return to work was determined by documentation from treating physician. If patient had returned to work at any point in study period, they were documented in the affirmative. RTW is presented with an associated 95 % exact binomial confidence interval. Logistic regression was used to assess the association of patient age, gender and number of injections with RTW in the patients who received ESI treatment. Statistical analyses were performed using SAS v9.4 (SAS Institute, Inc. Cary NC USA).

## 3 Results

The electronic medical data review identified 6815 patients who possessed the combination of qualifying ICD 9/10 diagnostic codes and epidural procedural CPT codes. The cohort was stratified into workers compensation and non-workers compensation classes. 563 patients were placed in the workers compensation class based on inclusion criteria. Of the 563 patients, 247 patients were not working at the time of treatment with a lumbar ESI. The remaining 315 patients worked at the time of injection and continued to work following the injection(s). Focusing on the 247 patients out of work at time of treatment with ESI, 152 were male (61.5 %). 222 (89.9 %) patients received at least one lumbar ESI, and 92 (41.4 %) patients received three or more injections for treatment. Twenty-five patients in the cohort of 247 were excluded from analysis following manual review of the patients’ medical record (resulting in 222). Reasons for exclusion included: ESI ordered but never performed, insufficient chart information to determine if/when a patient returned to work. 23 of 222 patients treated with a lumbar ESI returned to work, this totaled 10.4 % of the subgroup (95 % CI: 6.7 %–15.1 %). There was insufficient evidence of an association between gender and return to work following treatment with a lumbar ESI (OR for male = 1.09, 95 % CI 0.44–2.69, p = 0.86). Similarly, there was no association between the number of injections and return to work (OR for 3+ injections vs 1–2 injections = 0.73, 95 % CI 0.30–1.80, p = 0.49). However, we observed a decrease in likelihood of returning to work after ESI injection as age increases (OR per 10 years of age = 0.51, 95 % CI 0.36–0.73, p = 0.0002).

## 4 Discussion

Our study observed the return-to-work rate after treatment with a lumbar ESI was 10.4 %. The number of injections, age and gender of the patient seemed to have no effect on the rate of return to work. Our data suggest that use of a lumbar ESI may not provide the intended benefit of pain relief for the purpose of returning to work. A previous study also supported the idea that lumbar ESI had no effect on pain or function compared to treatment of pain without an ESI [10].

The desire to carry out this study originated from our clinical experience that showed injured workers with lumbar radiculopathy did not respond to treatment with a lumbar ESI compared to non-work-related patients. This impression is supported by prior studies evaluating the outcome of surgery to treat lumbar radiculopathy in work versus non-work-related patients. [11,12,13]. A 2023 study found that patients who were designated as workers compensation (WC) status with a diagnosis of LBP who underwent surgery had higher rates of disability, increased postoperative pain, lower quality of life, and delayed return to work (RTW) compared to patients who were not designated as WC cases [11]. Regarding lumbar ESI and return to work, Warfield et al. found that patients with compensation claims were less likely to have a positive effect with ESI compared to non-compensation patients [14] A 2024 study observed a 61 % return to work rate within four weeks of receiving an injection. The study did not describe whether patients were injured workers or managed through a workers compensation system [15]. All the patients in our system were injured workers, and the lumbar radiculopathy was a result of a work injury.

Previous systematic reviews of lumbar ESI determined short term improvements of pain and function, possible avoidance of spinal surgery and lower cost of care without specific mention of return to work [[4], [5], [6],^16^]. Two commonly used disability research scales do not specifically inquire about return to work [17,18]. Medical treatment guidelines developed for the evaluation and management of injured workers emphasize return to work as a criterion for improved physical function [8,9]. Insurance carriers potentially use return to work as a requirement for authorizing treatment of lumbar radiculopathy. All injured workers are managed in a similar algorithmic method based on state issued medical treatment guidelines [8]. Anti-inflammatory medication, physical therapy or chiropractic care are offered as initial treatment to all workers with lumbar radiculopathy. Lumbar ESI is offered when initial treatment fails to improve pain and function, especially their ability to return to work.

Our study has limitations that we will discuss at this time. The retrospective design limits our ability to know what confounders effected the patients in real-time that contributed to our RTW rate (10.4 %). A randomized controlled study is more suited to evaluate our hypothesis but more challenging to complete as injured workers receive evaluation, treatment and financial compensation from government funded sources. The retrospective study did not allow control of several factors that influence a patient's ability to return to work including availability of accommodated work, financial incentive to return to work, monetary compensation available while out of work and job satisfaction. We could not control for additional types and duration of treatment (physical therapy, chiropractic care, medication) used for lumbar radiculopathy. However, these variables may have been sufficiently distributed among all patients evaluated in our study.

Despite these limitations, our study suggests a dismal return-to-work rate for injured workers treated with an epidural steroid injection. Our results question whether lumbar ESIs should be offered to workers for treatment of lumbar radiculopathy. Lumbar epidural steroid injections are costly, associated with higher patient morbidity and place additional administrative burden on patients and clinicians to obtain authorization for the procedure. Lumbar ESIs may provide short term improvement in pain or increase value of care by reducing the need for spine surgery like the non-worker compensation population but specific studies are necessary to assess these issues. Tracking of workers compensation patient outcomes or use of a patient registry would be useful steps to determine the benefit and cost effectiveness of treatment of lumbar radiculopathy. We hope our study adds to the limited literature on treatment outcomes with injured workers.

## 5 Conclusion

Return to work rates in WC patients diagnosed with lumbar radiculopathy was 10.4 %. Further studies to determine return to work rates in a prospective manner with comparison groups are warranted given ESIs are a mainstay of treatment for lumbar radiculopathy in injured workers.
